# Economic evaluation of human papillomavirus vaccination in the United Kingdom

**DOI:** 10.1136/bmj.a769

**Published:** 2008-07-31

**Authors:** Mark Jit, Yoon Hong Choi, W John Edmunds

**Affiliations:** 1 Modelling and Economics Unit, Centre for Infections, Health Protection Agency, London NW9 6BT

## Abstract

**Objective** To assess the cost effectiveness of routine vaccination of 12 year old schoolgirls against human papillomavirus infection in the United Kingdom.

**Design** Economic evaluation.

**Setting** UK.

**Population** Schoolgirls aged 12 or older.

**Main outcome measures** Costs, quality adjusted life years (QALYs), and incremental cost effectiveness ratios for a range of vaccination options.

**Results** Vaccinating 12 year old schoolgirls with a quadrivalent vaccine at 80% coverage is likely to be cost effective at a willingness to pay threshold of £30 000 (€37 700; $59 163) per QALY gained, if the average duration of protection from the vaccine is more than 10 years. Implementing a catch-up campaign of girls up to age 18 is likely to be cost effective. Vaccination of boys is unlikely to be cost effective. A bivalent vaccine with the same efficacy against human papillomavirus types 16 and 18 costing £13-£21 less per dose (depending on the duration of vaccine protection) may be as cost effective as the quadrivalent vaccine although less effective as it does not prevent anogenital warts.

**Conclusions** Routine vaccination of 12 year old schoolgirls combined with an initial catch-up campaign up to age 18 is likely to be cost effective in the UK. The results are robust to uncertainty in many parameters and processes. A key influential variable is the duration of vaccine protection.

## Introduction

Human papillomaviruses are responsible for nearly 3000 cases of cervical cancer^1^ and more than 100 000 diagnosed cases of anogenital warts^2^ in the United Kingdom every year, despite a decrease in the incidence of cervical cancer as a result of regular cytological screening. In particular, human papillomavirus types 16 and 18 are associated with 70% of cervical cancers,^3^ whereas 90% of anogenital warts are linked to human papillomavirus types 6 and 11.^4^ In addition, human papillomavirus has been linked to anal, vulval, vaginal, penile, and oropharyngeal cancers.^5^ Two prophylactic vaccines against human papillomavirus (a bivalent vaccine against types 16 and 18 and a quadrivalent vaccine that also includes types 6 and 11) have been shown to be efficacious in up to five years of follow-up against types 16 and 18 cervical infection and associated disease as well as against anogenital warts.^6^ ^7^ In addition, the results from clinical trials suggest that both vaccines may offer partial protection against oncogenic human papillomavirus types not in the vaccine.^7^ ^8^ Both vaccines have the potential to bring a decrease in the incidence of human papillomavirus related disease and are being considered for routine immunisation in many countries. Information about the epidemiological and economic impact of adding vaccination to the cervical screening system is required for a decision to be made about whether and how to introduce vaccination.

In the UK the Department of Health has announced a routine human papillomavirus immunisation programme for schoolgirls aged 12 or 13, starting from September 2008, with a two year catch-up programme for girls up to 18.^9^ The announcement follows advice from the Joint Committee on Vaccination and Immunisation, which considered the cost effectiveness of different options such as vaccinating at different ages, extending vaccination to boys, and incorporating a catch-up campaign for girls older than the age for routine vaccination. The same cost effectiveness model was later used to inform the adjudication process between the two vaccines, which led to a decision to use the bivalent vaccine in the UK immunisation programme.

We describe the cost effectiveness model used to inform decisions about human papillomavirus vaccination in the UK. Our model considers the impact of vaccination on squamous cell carcinomas, adenocarcinomas, cervical cancers due to high risk human papillomavirus types not in the vaccine, non-cervical cancers, and anogenital warts.

## Methods

### Model

We used a transmission dynamic model to predict the burden of human papillomavirus related disease for the number of cervical screens, treatments for precancerous abnormalities of the cervix, and cases of diagnosed cancer and anogenital warts expected before and after vaccination.^10^ The model incorporated sexual transmission to take into account the additional benefit of herd immunity. The population was stratified by papillomavirus type, age, sex, and sexual activity based risk group. Papillomavirus types were split into three groups for cervical cancers (type 16, type 18, and other high risk types) and two groups for anogenital warts (type 6 and type 11).

For high risk papillomavirus types causing squamous cell carcinomas, we used type specific model compartments to represent being susceptible to infection, infected with human papillomavirus or immune to infection. Infected states in females were subdivided into being free of disease, having cervical intraepithelial neoplasias of different grades (1, 2, or 3), having carcinoma in situ or undiagnosed squamous cell carcinoma and having diagnosed invasive squamous cell carcinoma. We adopted the same structure for adenocarcinomas, with states for cervical glandular intraepithelial neoplasia replacing cervical intraepithelial neoplasia. Infected males could only occupy a single disease free state. For human papillomavirus types 6 and 11 we used three type specific compartments for both sexes, representing being susceptible to infection, being infected, and being immune to human papillomavirus infection.

People in infected states could regress to less severe disease states or to the immune state as a result of natural regression or cervical screening followed by treatment. Natural immunity could wane, causing people to again be susceptible to infection of the same type. We used a range of possible values for the rate at which infection was cleared and the rate at which natural immunity was lost. Progression and regression rates were determined by fitting models to data on prevalence of human papillomavirus, results for cervical screening, and diagnoses of cervical cancer. The vaccines have shown close to 100% efficacy against clinical end points in females naive to human papillomavirus.^11^ The model simplifies these results by assuming that vaccine protected people have 100% protection against any vaccine type human papillomavirus infection.

We constructed a total of 2700 possible scenarios for high risk papillomavirus types (or groups of types) and 900 scenarios for low risk types from combinations of assumptions on the epidemiology of human papillomavirus, clinical course of infection, accuracy of cervical screening, and rates of sexual partnership. Of these we chose outcomes from 72% of scenarios for the economic analysis on the basis of their goodness of fit to prevalence data on human papillomavirus.^12^ We then generated parameter values for cost and utility weights by Monte Carlo sampling (using the Latin hypercube method) from the joint distribution of plausible ranges of values for each parameter (see table 1). To estimate the cost to the health service and benefits gained we combined each set of economic parameters with one of the transmission dynamic model scenarios. By sampling from different combinations of epidemiological and economic assumptions we constructed a total of 50 000 meta-scenarios.

**Table 1 tbl1:** Parameters used in economic model

Parameter	Distribution	Reference
Total cost (£):		
Cervical screen	Normal mean 55 (SD 4.4)	18; 13
Colposcopy	Normal mean 216 (SD 81)	20
Treatment for precancerous lesions*	Normal mean 332 (SD 27)	19; 20
Lifetime cost of cancer treatment		
Stage I	Lognormal based on normal mean 9.30 (SD 0.63)	28
Stage II	Lognormal based on normal mean 9.68 (SD 0.82)	28
Stage III	Lognormal based on normal mean 9.84 (SD 0.50)	28
Stage IV	Lognormal based on normal mean 9.53 (SD 1.01)	28
Treatment for warts	Normal 134 (SD 9.08)	34
Vaccine cost (per dose)	Uniform 60.00, 80.50	Market price
Administration cost (per dose): school based	Normal mean 4.37 (SD 1.09)	15
Administration cost (per dose): general practitioner based	Normal mean 10 (SD 2.5)	13
Total QALY detriment:		
Positive Pap smear result received	Normal mean 0.03 (SD 0.015)	24; 26; 27
Colposcopy result received		
Cervical intraepithelial neoplasia grade 1	Normal mean 0.012 (SD 0.031)	24; 26; 27
Cervical intraepithelial neoplasia grade 2	Normal mean 0.065 (SD 0.0051)	24; 26; 27
Cervical intraepithelial neoplasia grade 3	Normal mean 0.054 (SD 0.051)	24; 26; 27
Year of treatment		
Cancer stage I	Normal mean 0.35 (SD 0.082)	29
Cancer stage II	Normal mean 0.44 (SD 00.71)	29
Cancer stage III	Normal mean 0.44 (SD 0.071)	29
Cancer stage IV	Normal mean 0.52 (SD 0.061)	29
Year of post-treatment		
Cancer stage I	Normal mean 0.10 (SD 0.066)	29
Cancer stage II	Normal mean 0.15 (SD 0.077)	29
Cancer stage III	Normal mean 0.15 (SD 0.077)	29
Cancer stage IV	Normal mean 0.38 (SD 0.061)	29
Warts diagnosed	Normal mean 0.039 (SD 0.017)	32
Sensitivity for cervical intraepithelial neoplasia grade 1	Triangular 0.17, 0.69, 0.99	23
Specificity for cervical intraepithelial neoplasia grade 1	Triangular 0.09, 0.81, 1.00	23
Sensitivity for cervical intraepithelial neoplasia grades 2 or 3	Triangular 0.23, 0.83, 1.00	23
Specificity for cervical intraepithelial neoplasia grades 2 or 3	Triangular 0.21, 0.92, 1.00	23
Years after treatment with reduced life quality	Triangular 0, 5, 100	Assumed
Multiplier for cancer survival rates	Normal mean 1.00 (SD 0.022)	29
Duration (days) of anogenital warts episode	Normal mean 52.6 (SD 5.08)	31
Proportion of girls receiving only 1 dose	Normal mean 2 (SD 0.5)	14
Proportion of girls receiving only 2 doses	Normal mean 9 (SD 2.25)	14

QALY=quality adjusted life years. £1.00 (€1.26; $1.98).

*Cervical intraepithelial neoplasia grades 1, 2, or 3.

We followed annual birth cohorts of 80 000 infants in the transmission model for 100 years after the onset of vaccination, with costs inflated to 2006-7 prices (in sterling) according to the hospital and community health services pay and price inflation index.^13^ Uninflated costs are presented in the text but are inflated to 2006-7 prices in table 1. We measured health utilities in quality adjusted life years (QALYs), discounted costs and benefits at 3.5% per year in the base case, and adopted a healthcare provider perspective on costs, as required by the National Institute for Health and Clinical Excellence.^14^

### Vaccination strategies

We analysed the cost effectiveness of a range of vaccination strategies. The base case assumption was vaccination of girls aged 12 years using a school based programme, with 80% vaccine coverage (just under 400 000 girls) for the full three doses, on the basis of reported coverage in a trial of a school based hepatitis B vaccination programme.^15^ A pilot study in schools in Manchester found the uptake of vaccine against human papillomavirus to be as low as 70%,^16^ which we considered in an alternative scenario. A proportion of girls were assumed to receive only one or two doses, on the basis of reported figures from the hepatitis B study.^15^ We assumed that those receiving fewer than three doses received no protection against human papillomavirus. Alternative scenarios were vaccinating girls at ages 13 or 14 instead, vaccinating boys and girls at age 12, a catch-up campaign in the first year of vaccination to vaccinate females from age 12 to ages 14, 16, 18, or 25, and achieving a coverage of 70% or 90% for the full three doses.

We analysed the cost effectiveness of two vaccines: a vaccine that protects against human papillomavirus types 6, 11, 16, and 18, and a vaccine that protects against types 16 and 18 only (therefore does not protect against anogenital warts). Analysis of each vaccination strategy included the impact of vaccination on the incidence of neoplasias, cervical cancer, and warts in females and of warts in males. All base case analyses used the quadrivalent vaccine. We assumed that vaccinating people infected with human papillomavirus would have no effect on disease progression but would protect them from subsequent infection, as suggested by vaccine trials.^6^ Results from clinical trials of the quadrivalent vaccine indicate that the vaccine protects women for at least five years,^17^ but epidemiological evidence beyond that time scale is lacking. We therefore assumed the proportion of protected vaccinated women declined exponentially from the time of vaccination, with the average duration of protection varying between life long and 10 years.

To ensure reliable comparisons between strategies we used the same set of 50 000 meta-scenarios to model each vaccination strategy.

### Economic parameters

#### Screening

Cervical screening in the UK is currently carried out using the Pap smear, although this is being replaced by liquid based cytology. We therefore estimated the cost of a screen as the midpoint between the costs of a conventional Pap smear (£21.70,^18^ 2002 prices) and liquid based cytology (£25.40,^19^ 2003 prices). We added the costs of consultations with a general practitioner (£2.50 per minute,^13^ 2006 prices) on the basis of average consultation times of 13.3 minutes for a Pap smear and 8.6 minutes for liquid based cytology.^18^ Costs for screening at sexual health clinics and outpatient clinics are higher (£37 20 and £68^19^ respectively, 2003 prices), but we did not use them as they account for under 10% of screens.^21^ We assumed the cost of a colposcopy to be normally distributed, with distribution parameters following standard NHS reference costs.^22^

We estimated the number of screens and colposcopies per woman on the basis of the UK screening protocol, and estimates of sensitivity and specificity of the Pap smear.^23^ The sensitivity and specificity of the Pap smear were varied over the range suggested by Nanda et al.^23^ The range is wide enough to encompass possible improvements resulting from the introduction of liquid based cytology. Estimates of the quality of life detriment while waiting for follow-up after a positive Pap smear result or a positive colposcopy result were based on questionnaires given to healthy volunteers.^24^ We assumed the waiting period for a follow-up Pap smear to be six months and for a colposcopy one month, as recommended by guidelines from the NHS cervical screening programme.^25^ If a woman received a positive colposcopy result we assumed that she would wait 1.6 months for follow-up after a cervical intraepithelial grade 1 result, 0.6 months after a grade 2 result, and 5.0 months after a grade 3 result.^26^ We sampled quality of life detriment during this time from a normal distribution centred around time trade-off estimates from a survey of university students.^24^ We fixed the variance to ensure that much lower estimates of quality of life detriment using committee consensus health utility index estimates from an Institute of Medicine report^27^ would be the lower 95% centile of the distribution. Treatment costs after a positive colposcopy result ranged from £280 (2004 prices) per treatment for large loop excision of the transformation zone using data from private insurers’ fee schedules^20^ to £329 (2004 prices) per treatment for cone biopsy or large loop excision of the transformation zone using NHS reference costs.^19^ We used these two results to estimate the mean and variance of a normally distributed parameter for the relevant treatment cost.

#### Cancer

We stratified the average treatment costs^28^ and quality of life weights^29^ associated with treatment for each stage of cervical cancer by age at time of detection according to the classification given by the International Federation of Gynecology and Obstetrics. We used age groups of 10-49, 50-69, and 70 or more; these correspond to those used in a Dutch study of survival rates in patients with cervical cancer.^30^ Mortality rates for patients aged 50 or more were increased by 12.5% to account for reported differences in survival from cervical cancer in older patients between the Netherlands and England.^31^ We assumed treatment for cervical cancer to last five years unless the patient died before that time. To determine the number of years between a diagnosis of cancer and death in non-survivors we used five year survival curves.^30^ Rates were varied in the sensitivity analysis within their reported 95% confidence intervals. We assumed that patients who survived beyond five years had a normal life expectancy but reduced quality of life.^29^ The length of time spent in this state of reduced quality of life was assumed to vary between no years to the remaining lifetime of the recovered patient. We sampled quality of life detriments associated with patients and survivors of cancer from normal distributions fitted to the base case and range used in a previously published model.^29^ Healthcare costs of treating patients with cancer are available from an audit of the Trent Cancer Registry^28^; we fitted the reported means and standard deviations to a lognormal distribution. We assumed that costs and quality of life detriments for non-cervical cancers were the same as for cervical cancers.

#### Warts

Quality of life of patients with anogenital warts was estimated on the basis of data from a cross sectional survey of 81 attendees to a sexual health clinic in York using the EQ-5D questionnaire.^32^ We did not use other published data as they were based on interviews with healthy volunteers or doctors^24^ ^27^ or on studies with small sample sizes.^33^ The cost of treating an episode of anogenital warts was £134 (2006 prices), estimated from an audit of 189 case notes in the same clinic.^34^ We included new and recurrent cases in the analysis. This is slightly lower than previous estimates in the literature, which range between £180^35^ and £191^20^ when adjusted to 2007 prices. We used the same notes to estimate the length of an episode of warts, defined as the time from first attendance for warts and ending when a patient was either declared free of warts, did not subsequently return after being instructed to return only if warts recurred, or was lost to follow-up. We assumed that patients lost to follow-up after only a single visit had an episode length of 28 days (the average length of a prescription for home treatment for warts given by the clinic). Patients who still had warts at the end of the study period were censored. We added an additional seven days to the length of each episode of warts to represent the time between the appearance of symptoms and attendance at the clinic.

#### Vaccination costs

We varied the cost for a dose of the quadrivalent vaccine between £60 (catalogue price of Gardasil in the United States; Merck, NJ) and £80.50 (price at which Gardasil is currently available privately in the UK^36^; Sanofi Pasteur MSD, Berks). The cost of administration was estimated at £3.56 per dose (2001 prices), based on the estimated administration cost of hepatitis B vaccination by a school based programme.^15^ For catch-up campaigns, females aged over 16 years were assumed to receive their vaccination through general practice clinics and hence instead incur a cost of £10 (2006 prices) for a nurse consultation.^13^

### Alternative scenarios

#### Vaccinating unscreened women

The transmission model considers the effect of human papillomavirus vaccination in a screened population. We considered in a separate scenario estimates of vaccine impact on cancers in unscreened women by setting screening rates to zero. The size of the unscreened population was assumed to be 3.8% of the entire cohort of females eligible for screening, based on the proportion of women aged 45-49 years estimated to have never attended screening.^21^ To estimate the maximum possible benefit from vaccination, we assumed that a human papillomavirus vaccine reduces the incidence of cancer in unscreened women in a particular age group by the median proportion it reduces the incidence of cancer in screened women in the same age group. This is an overestimate as it assumes that the maximum reduction occurs immediately after vaccination and that cervical cancers occur at the same age in screened and unscreened women.

#### Vaccine impact on non-cervical cancers

Worldwide, oncogenic human papillomavirus types may be linked to 90% of anal cancers, 40% of vulval, vaginal, and penile cancers, and 12% of oropharyngeal cancers.^5^ The quadrivalent vaccine has shown good efficacy against vulval and vaginal neoplasias in clinical trials,^37^ and it is possible that the vaccines would be effective against non-cervical cancers caused by vaccine type human papillomavirus. We therefore estimated the additional benefit of assuming that both vaccines are fully effective against vaccine type infections leading to non-cervical cancers. The annual incidence of such cancers was obtained from the Office for National Statistics,^1^ whereas the proportion of these cancers attributable to human papillomavirus types 16 and 18 was obtained from a seroprevalence study among patients with anogenital cancer.^38^ We did not include estimates in the base case because of the uncertainty about whether vaccination fully protects against all these cancers and the lack of reliable information about the fraction attributable to each human papillomavirus type and the progression to disease.

#### Vaccine impact on non-vaccine types

In the base case we assumed that vaccination afforded no protection against non-vaccine type human papillomavirus infection. Some protection against non-vaccine types has been suggested in clinical trials of both vaccines, however.^7^ ^8^ We considered the additional reduction in cervical cancer incidence of cross protection with an efficacy of 27% against oncogenic non-vaccine types as suggested in the literature.

### Analysis of individual parameter contribution

We analysed the relative contribution of each epidemiological and economic parameter on the incremental cost effectiveness of vaccination. Epidemiological scenarios were converted into integer codes. For example, the duration of natural immunity was coded as 0 for none, 1 for three years, 2 for 10 years, 3 for 20 years, and 4 for life long. We used each epidemiological scenario and each economic parameter in table 1 as a predictor variable in a linear regression model, with the cost per QALY gained as the outcome variable. To correct for heteroskedasticity we used robust standard errors. We chose parameters in the model by using backward stepwise regression with a significance cut-off of 0.05. Each coefficient was then multiplied by the end points of the corresponding 95% confidence interval for the parameter (based on the distributions shown in table 1) to obtain a predicted range for the cost effectiveness ratio as each parameter was varied.

### Price differential for quadrivalent and bivalent vaccines

For the base case scenario we determined the estimated price differential between equally cost effective bivalent and quadrivalent vaccines. The price differential was calculated by dividing the net present value of the additional benefits of a quadrivalent vaccination programme for preventing anogenital warts (cost savings to the health service and quality of life gains) by the number of doses of vaccine required to achieve the benefits. The net present value was calculated on the basis of a willingness to pay threshold of £30 000 per QALY gained, based on the perception that this is approximately the cost effectiveness threshold used for evaluating health technologies in the UK.^39^

## Results

The median cost per QALY gained of the base case scenario (vaccinating girls aged 12 at 80% coverage with a quadrivalent vaccine) with vaccine protection for an average of 20 years is £22 500 (95% range £13 800-£32 900). The median net discounted cost of the programme over 100 years (cost of vaccination minus cost to the health service saved due to reduction in human papillomavirus related outcomes) is £1.8bn (95% range £1.5bn-£2.2bn, table 2).

### Epidemiological impact of vaccination

Model results^10^ predict that vaccinating 12 year old girls in a screened population at 80% coverage with a quadrivalent human papillomavirus vaccine that protects for an average of 20 years reduces the incidence of cervical cancer by 24-93% and anogenital warts by 22-100% after 100 years of an ongoing vaccination programme. Catch-up campaigns reduce incidence in the first 30 years of vaccination but have little effect beyond that. Extending vaccination to boys provides only a small additional reduction in incidence of cervical cancer and anogenital warts, with the greatest benefit when the duration of vaccine protection is short.

### Cost effectiveness

Table 2 shows the cost and QALYs gained by the introduction of vaccination, over the 100 years after the introduction of the vaccine, assuming base case assumptions (vaccination of girls at age 12, 80% coverage, quadrivalent vaccine, and no catch-up programme) and vaccine protection lasting on average 20 years. The vaccination programme is expected to generate cost savings to the health service from reduced treatment. These are, however, outweighed by the cost of the vaccination programme itself, estimated to be £77m (95% range £68m-£88m) a year. Hence vaccination is unlikely to be cost saving. QALYs are gained through a reduction in the detection and treatment of cervical dysplasia (or presumed cervical dysplasia), anogenital warts, and cervical cancer. Roughly three quarters of the discounted QALYs gained result from a reduction in the incidence of cancer. A reduction in the incidence of warts contributes to about a 10th of discounted QALYs gained but to about half of the discounted cost savings to the health service.

**Table 2 tbl2:** Discounted costs and quality adjusted life years (QALYs) gained by human papillomavirus vaccination in base case programme (vaccination of girls aged 12 years with no catch-up programme, 80% effective vaccine coverage, quadrivalent vaccine, 100 year time horizon, and 3.5% discount rate for costs and benefits) assuming vaccine protection lasts an average of 20 years

Parameter	Mean	Median	5% centile	95% centile
Cost of vaccine (£m)	2225	2225	1937	2513
Cost saved (£m):				
Reduced lesion screening and treatment	−48	−47	−176	61
Cancers prevented	−135	−118	−274	−55
Warts prevented	−229	−232	−300	−151
Net cost (£m)	1813	1816	1455	2169
Cost saved (£m):				
Unscreened vaccinees	−16	−14	−34	−7
Non-cervical cancers prevented	−42	−34	−96	−16
Non-vaccine type infections prevented	−61	−55	−110	−34
Net cost with unscreened, non-cervical cancers, and non-vaccine type infections (£m)	1694	1701	1288	2074
QALYs gained:				
Reduced lesion screening and treatment	10 987	9 617	−783	27 607
Cancers prevented	63 353	60 976	44 326	90 051
Warts prevented	9530	9233	4434	15 711
Total QALYs gained	83 871	80 690	58 856	119 167
QALYS gained:				
Unscreened cohort	7444	6778	5048	12 137
Non-cervical cancers prevented	18 033	15 171	11 085	34 924
Non-vaccine type infections prevented (£m)	8799	7989	5378	15 531
Total QALYs gained with unscreened, non-cervical cancers, and non-vaccine type infections	118 146	111 304	82 874	177 199

£1.00 (€1.26; $1.98). Mean, median, 5% and 95% centiles of 50 000 meta-scenarios representing uncertainty in epidemiological and economic parameters are reported.

Table 2 also shows the additional cost savings and QALY gains from protecting the unscreened population, protecting against non-cervical cancers linked to human papillomavirus, and partially protecting against infection by non-vaccine human papillomavirus types. Of the three categories, the QALY benefit from preventing non-cervical cancers is greatest. However, the figures assume these cancers follow a similar progression to cervical cancers. Partial protection against infection by non-vaccine human papillomavirus types has the greatest potential for cost savings, as these types account for a large portion of positive cervical smear results that then require expensive follow-up procedures. The QALY gains from preventing these infections are smaller because these human papillomavirus types account for a minority (about 30%) of cervical cancers in the UK.

Figure 1 shows cost effectiveness acceptability curves for this vaccination programme, given different assumptions about the duration of protection from the vaccine. The results suggest that if vaccine induced immunity is short lived (10 years on average) then it is unlikely that vaccination of 12 year old girls would be acceptable on the grounds of cost effectiveness (a small proportion of the simulations result in a cost per discounted QALY gained of less than £20 000-£30 000). If the vaccine induces lifelong protection, however, then more than 80% of scenarios suggest that vaccination would be regarded as cost effective (below £20 000-£30 000 per QALY gained). If vaccine induced immunity lasts on average around 20 years then about 25-85% of scenarios suggest that the vaccination programme is cost effective (fig 1). Figure 1 also shows cost effectiveness acceptability curves resulting from the use of more optimistic assumptions. Assuming that vaccine uptake is 80% in girls who do not subsequently attend cervical screening is not sufficient to make a vaccine with 10 years’ protection cost effective. If this assumption is used and the vaccine is also assumed to be efficacious against non-cervical cancers and partially efficacious against infection by non-vaccine human papillomavirus types, then vaccination may be cost effective even if vaccine induced immunity lasts only 10 years on average.

**Figure fig1:**
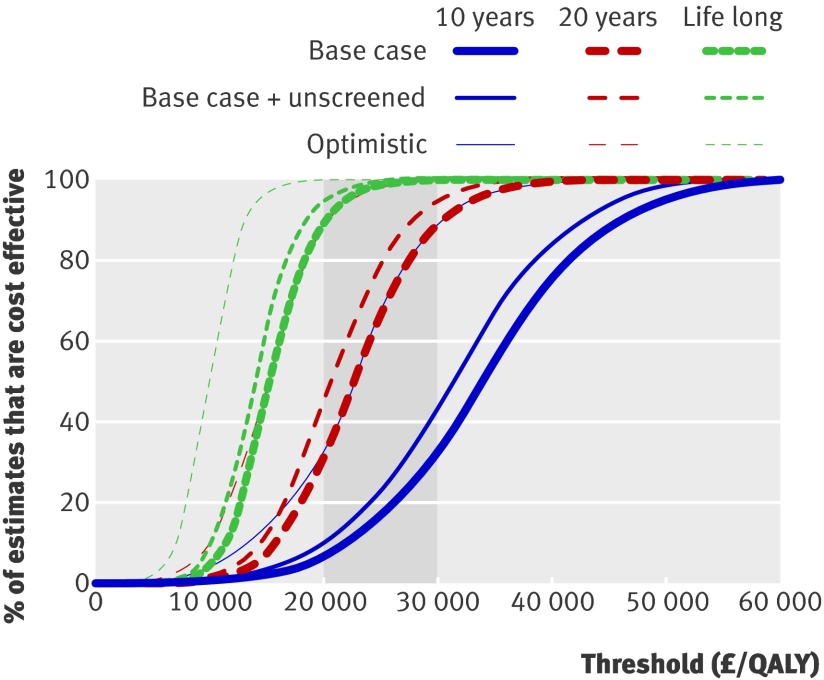
**Fig 1** Cost effectiveness acceptability curves for base case vaccination programme (girls aged 12 years only, quadrivalent vaccine, 80% coverage) under different assumptions about duration of protection from vaccine. Incremental cost effectiveness of vaccination compared with no vaccination option is shown. Region of £20 000- £30 000 per QALY gained is shaded. Thick solid and dashed lines indicate cost effectiveness acceptability curves when considering vaccine type cervical cancers in a screened population, medium solid and dashed lines indicate curves assuming 80% coverage in screened and unscreened populations, and thin solid and dashed lines indicate curves assuming 80% coverage in screened and unscreened populations, protection against non-cervical cancers, and some cross protection against non-vaccine types

### Age at vaccination

Figure 2 shows that offering vaccination to girls aged 13 or 14 instead of 12 (as in the base case) increases the likely cost effectiveness of the programme, particularly if vaccine induced immunity is not life long. This is for two reasons: firstly, vaccinating at a slightly older age means that the delay to benefits being accrued is shorter and as health benefits are discounted this more rapid accrual of benefits is preferred. Secondly, if vaccine induced immunity is relatively short lived (10-20 years on average), and declines exponentially (as assumed) then delaying vaccination for a few years results in greater protection for women during the highest risk period for acquisition of human papillomavirus (late teens and 20s). It has not, however, been assumed that there are increased costs or a drop-off in coverage associated with vaccinating at a later age. Also, these findings depend on the low prevalence of human papillomavirus infection before age 14 in the UK^40^ and are not generalisable to countries in which sexual activity starts at an earlier age. The additional benefit of delaying vaccination by one or two years of life is most clearly seen for reduction in the incidence of warts, because this peaks in the early 20s and because the best fitting scenarios for warts related human papillomavirus types (6 and 11) are associated with a short duration of natural immunity. Extending vaccine protection by just a few years can therefore have a large effect on the acquisition of wart related human papillomavirus types.

**Figure fig2:**
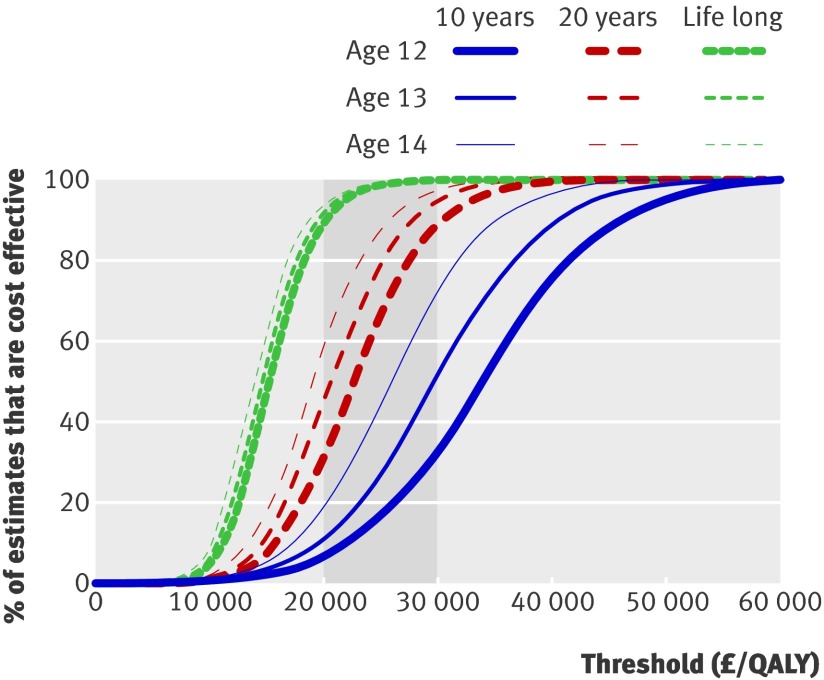
**Fig 2** Cost effectiveness acceptability curves for vaccination of girls aged 12, 13, or 14 years with a quadrivalent vaccine at 80% coverage, under different assumptions about vaccine duration of protection, based on results of 50 000 meta-scenarios combining epidemiological and economic assumptions. Incremental cost effectiveness of vaccination compared with no vaccination option is shown. Region of £20 000-£30 000 per QALY gained is shaded

### Vaccine coverage

The cost per QALY gained of vaccination is insensitive to vaccine coverage in girls over the range explored (70-90%). This is because there is an approximately linear relation between coverage and the reduction in disease as well as a linear relation between coverage and the costs of the programme. Although greater coverage increases the costs and benefits of the programme it does not significantly alter the cost per QALY gained (fig 3). The effect of reducing coverage to below 70% was not explored. Models of vaccination against human papillomavirus in developing countries^41^ ^42^ suggest that levels of coverage around 50% may be more cost effective than higher levels of coverage, perhaps because of the increased benefit from herd immunity.

**Figure fig3:**
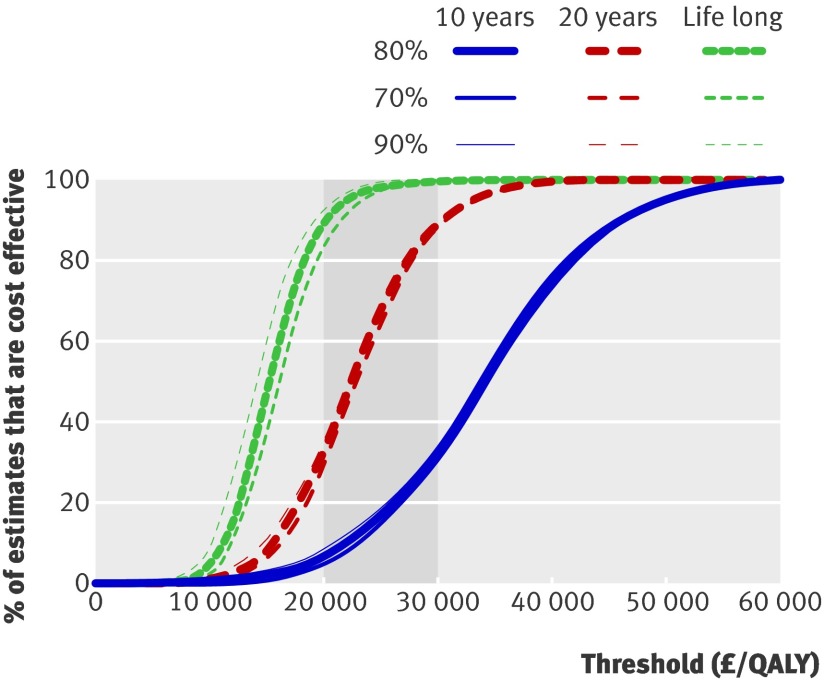
**Fig 3** Cost effectiveness acceptability curves for vaccination of girls aged 12 years with a quadrivalent vaccine at different levels of three dose coverage, for different assumptions about duration of protection from vaccine. Incremental cost effectiveness of vaccination compared with no vaccination option is shown. Region of £20 000-£30 000 per QALY gained is shaded

### Vaccination of boys

Vaccination of boys at age 12 years in addition to girls is unlikely to be cost effective, even if vaccination results in lifelong protection (table 3). This is because at 80% coverage it is likely that most cervical cancers due to human papillomavirus types 16 and 18 will be prevented along with many cases of anogenital warts (in both sexes). Therefore the additional benefits from vaccination of boys are few. This is particularly true if the duration of vaccine protection is long, as there is little additional burden to avoid.

**Table 3 tbl3:** Cost effectiveness of alternative human papillomavirus vaccination options of catch-up campaigns and vaccinating girls and boys, compared with base case vaccination option of vaccinating girls aged 12 years only

Programme	Average cost effectiveness ratio* (£)				Incremental cost effectiveness ratio† (£)		
	Median	5% centile	95% centile		Median	5% centile	95% centile
10 years’ vaccine protection:							
Girls aged 12	33 868	18 632	49 828		33 868	18 632	49 828
Catch-up aged 12-14	33 296	18 464	48 987		26 448	16 389	39 176
Catch-up aged 12-16	32 244	17 978	47 535		20 808	12 179	31 807
Catch-up aged 12-18	31 132	17 447	45 657		16 989	503	54 405
Catch-up aged 12-25	39 096	21 785	57 883		136 329	61 405	702 610
Girls+boys aged 12	53 099	31 696	77 780		113 846	71 099	176 749
20 years’ vaccine protection:							
Girls aged 12	22 474	13 722	32 920		22 474	13 722	32 920
Catch-up aged 12-14	22 210	13 636	32 553		18 856	12 296	27 992
Catch-up aged 12-16	21 789	13 420	31 996		16 417	10 238	25 117
Catch-up aged 12-18	21 126	13 061	30 898		11 856	Cost saving	31 107
Catch-up aged 12-25	27 432	17 069	40 327		128 302	64 003	557 772
Girls+boys aged 12	42 211	28 011	60 581		172 892	112 230	289 698
Lifetime vaccine protection:							
Girls aged 12	15 094	10 093	22 032		15 094	10 093	22 032
Catch-up aged 12-14	14 992	10 058	21 899		13 637	9297	20 493
Catch-up aged 12-16	14 877	10 004	21 760		13 204	8797	20 259
Catch-up aged 12-18	14 687	9912	21 453		11 509	2478	23 568
Catch-up aged 12-25	19 476	13 443	28 366		105 839	62 493	337 016
Girls+boys aged 12	33 281	23 814	46 994		520 255	304 798	986 917

£1.00 (€1.26; $1.98).

*Cost effectiveness of particular option compared with no vaccination option.

†Ratio of additional costs and benefits of particular vaccination programme compared with previous option. Options being compared with is no vaccination when considering routine vaccination of girls aged 12, routine vaccination of girls aged 12 when considering routine vaccination of both sexes aged 12, and programme listed immediately before for each of catch-up campaign options.

### Catch-up campaigns

Table 3 shows that a catch-up campaign in girls up to age 18 may be cost effective, but extending the campaign to age 25 is highly unlikely to be cost effective. Campaigns up to age 18 are more likely to be cost effective than the base case programme, because vaccinating at a slightly older age is more cost effective than vaccinating at age 12. This implies that if the base case programme is viewed favourably, then vaccinating girls up to age 18 in an initial catch-up campaign should also be adopted. Above this age an increasing proportion of females have previous evidence of human papillomavirus infection, and the delivery costs increase because it is assumed that vaccination in females older than 16 would be offered through their general practitioners rather than in a school based programme. A campaign up to age 18 actually weakly dominates shorter catch-up campaigns; however, shorter campaigns have been included in the analysis because it would be politically and operationally difficult to apply a combination of two strategies to the same population.^43^

### Price differential for quadrivalent and bivalent vaccines

Table 4 shows the estimated price differential between equally cost effective bivalent and quadrivalent vaccines for different assumptions about the vaccines’ average duration of protection. A bivalent vaccine would have to be around £13-£21 less expensive per dose for it to be as cost effective as an equivalent quadrivalent vaccine in a vaccination programme directed at 12 year old girls. The price differential is greatest if the vaccines both give lifelong protection. If the programme was extended with a catch-up programme for girls up to age 18, then the bivalent vaccine would have to be around £15-£23 less expensive per dose. Catch-up campaigns make the quadrivalent vaccine slightly more cost effective than the bivalent vaccine if the duration of vaccine protection is not life long, because they enable a larger portion of the burden of anogenital warts (which peaks in the early 20s) to be prevented.

**Table 4 tbl4:** Estimated price differential between equally cost effective bivalent and quadrivalent human papillomavirus vaccines (vaccination of girls aged 12 years, 80% effective vaccine coverage, 100 year time horizon, and 3.5% discount rate for costs and benefits)

Age at vaccination and catch-up	Duration of vaccine protection		
	10 years	20 years	Life long
Girls aged 12 only:			
Median (£)	12.96	17.27	20.94
95% centile (£)	22.06	26.59	30.30
5% centile (£)	6.26	9.37	12.95
Girls aged 12 with catch-up to age 18:			
Median (£)	15.54	20.28	23.21
95% centile (£)	25.26	29.91	32.79
5% centile (£)	8.58	12.28	14.98

£1.00 (€1.26; $1.98).

Results are based on 50 000 meta-scenarios representing uncertainty in epidemiological and economic parameters.

### Sensitivity analysis

Figure 4 shows the parameters in the linear regression model that produce the largest variation in the cost effectiveness ratio. The most important determinants of the cost effectiveness of the programme are the average duration of vaccine protection and of natural immunity. Vaccination has the greatest impact (and therefore is most likely to be cost effective) if the duration of protection is long and the duration of natural immunity is short. Table 5 shows the effect of altering the discount rate. The cost effectiveness ratio is highly sensitive to the discount rate for benefits because most of the QALY gains from preventing cervical cancers occur decades after the vaccination programme begins.

**Figure fig4:**
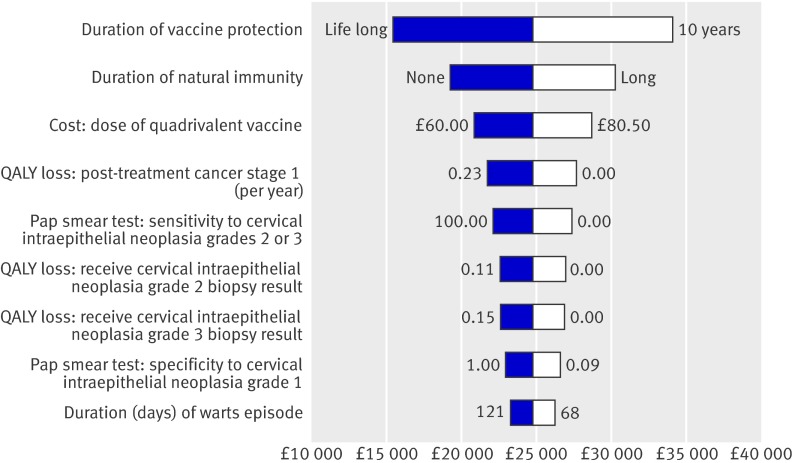
**Fig 4** Results of sensitivity analysis. Effect of changing each parameter over its range on estimated cost effectiveness of base case programme (estimates derived from regression model). 95% end points of range of each parameter are also shown

**Table 5 tbl5:** Effect of altering discount rate on incremental cost effectiveness ratio of base case programme (vaccination of girls aged 12 years, 80% effective vaccine coverage, 100 year time horizon, and 3.5% discount rate for costs and benefits)

Discount rate	Incremental cost effectiveness ratio (£)		
	Median	5% centile	95% centile
10 years’ vaccine protection:			
3.5% costs and benefits	33 868	18 632	49 828
5.0% costs and benefits	48 911	27 369	70 907
0% costs and benefits	11 273	5389	17 005
3.5% costs, 0% benefits	3446	1799	5084
6.0% costs, 1.5% benefits	5225	2887	7592
20 years’ vaccine protection:			
3.5% costs and benefits	22 474	13 722	32 920
5.0% costs and benefits	33 115	20 690	47 626
0% costs and benefits	7017	3692	10 762
3.5% costs, 0% benefits	2238	1339	3290
6.0% costs, 1.5% benefits	3495	2201	5032
Lifetime vaccine protection:			
3.5% costs and benefits	15 094	10 093	22 032
5.0% costs and benefits	22 785	15 617	32 470
0% costs and benefits	4320	2395	6794
3.5% costs, 0% benefits	1470	993	2132
6.0% costs, 1.5% benefits	2385	1685	3348

£1.00 (€1.26; $1.98).

Results are based on 50 000 meta-scenarios representing uncertainty in epidemiological and economic parameters.

## Discussion

Our economic analysis indicates that vaccinating 12 year old girls through a school based programme with a quadrivalent human papillomavirus vaccine priced at around £60-£80 per dose is likely to be cost effective at a threshold of £20 000-£30 000 per QALY gained as long as vaccine induced protection lasts more than 10 years. If the duration of protection is only 10 years then the programme may still be cost effective if high vaccine uptake can be achieved among girls who do not subsequently attend screening, if the vaccine is highly efficacious against non-cervical cancers caused by human papillomavirus types 16 and 18, and if it is partially efficacious against cervical cancers not caused by human papillomavirus types 16 and 18. Several parameters and model assumptions had a strong influence on the costs and health gains from human papillomavirus vaccination, in particular the duration of vaccine induced protection and the duration of natural immunity after infection with human papillomavirus. Results from the models also suggest that an initial catch-up campaign covering girls up to age 18 is likely to be cost effective, but vaccination of boys is unlikely to be cost effective.

### Strengths and weaknesses

A large number of possible model structures can be parameterised to match epidemiological data. Our approach involved an exhaustive search of this space of possible structures, fitting the models to available data, followed by sampling from the distribution of economic parameters. The range of models we have presented represent uncertainty in natural immunity to human papillomavirus infection; the sensitivity of the screening test; progression and regression between precancerous states; the prevalence of human papillomavirus types by age; the range of sexual partnerships in the UK population; and the impact of vaccination on other high risk types, the unscreened population, and non-cervical cancers.

Many other features of the biology of human papillomavirus are uncertain but were not varied as part of our uncertainty analysis. These include time dependent rates of loss of vaccine induced immunity and replacement of virus type by other human papillomavirus types after vaccination, as well as natural and vaccine induced cross immunity, synergy, or antagonism in progression rates for women infected with several types of human papillomavirus.

Only the 72% of model structures that best fit prevalence data for human papillomavirus were used in the analysis, but care should be taken when interpreting model results not to assume that each of the remaining model structures is equally plausible. Because of this the mean or median cost effectiveness ratio of the range of scenarios considered is not necessarily a reliable predictor of the most likely value for an actual vaccination programme. Instead, the entire range of results should be considered. In many cases most of the scenarios lie on one side of the £20 000-£30 000 per QALY gained threshold, indicating that reliable conclusions can be drawn about the cost effectiveness of a programme even if the more likely scenarios may be unequally distributed within the range of outputs.

### Comparison to other studies

To date only three published models have looked at the impact of vaccination in the UK.^44^ ^45^ ^46^ Two^44^ ^46^ are static models that do not consider the benefits of herd immunity. Thus they underestimate the cost effectiveness of human papillomavirus vaccination and cannot meaningfully estimate the cost effectiveness of vaccinating boys, as this is influenced by the impact that vaccinating girls alone would have on boys. One of the models^44^ does not present cost or utility implications of epidemiological outcomes. Also, existing models of human papillomavirus vaccination have either ignored adenocarcinomas^46^ or assumed that they behave in exactly the same way as squamous cell carcinomas.^44^ ^45^ We estimated the impact of vaccination on non-vaccine human papillomavirus types and considered the impact of vaccination on non-cervical cancers, which only a few recent models^47^ ^48^ have included. Both these benefits are important to consider as they have been claimed by the vaccine manufacturers.

Model predictions about the cost effectiveness ratio assuming lifelong vaccine induced protection are broadly in line with base case results from other published models of human papillomavirus vaccination in developed countries.^44^ ^45^ ^46^ ^47^ ^49^ ^50^ Our findings are likely to be more robust than most of these owing to our more exhaustive treatment of epidemiological uncertainty. Existing UK models^44^ ^45^ ^46^ are limited in terms of sensitivity analysis as they do not take full account of the many biological, epidemiological, and economic uncertainties surrounding human papillomavirus vaccination. Such models have assumed a base case structure and performed sensitivity analysis around the parameter values one or two at a time. For the case of human papillomavirus it is difficult to justify a choice of base case because of the uncertainty around many key parameters, and indeed the structure of the model itself. Only two published cost effectiveness models on human papillomavirus (from Canada^50^ and Brazil^41^ ^42^) have performed multivariate sensitivity analyses on model structure. Neither of these are transmission dynamic models, although the Brazilian model^41^ ^42^ uses post-vaccination incidences from a separate transmission model.

### Implications and future research

Cervical screening has significantly reduced the incidence of cervical cancer.^51^ Although we have included the estimated impact of screening in the model and tried to account for the accuracy of screening in our results, some of the details of the programme (such as rescreening women more often after a suspect result) are difficult to properly implement in the current model structure. In addition, although we attempted to estimate the impact vaccination may have on the unscreened population, accurate predictions of this depend on knowledge of who the unscreened population are (some are likely to be recent immigrants and therefore not reachable by a school based vaccination programme) as well as the possible rates of vaccine uptake in these groups. We found that the benefits of reaching unscreened groups can be significant. Since vaccination could act to either increase or decrease health inequalities depending on the uptake rates for screening and vaccination by socioeconomic groups and the level of herd protection generated, future modelling and surveillance work should deal with these aspects. Furthermore, our model assumes that the screening programme will continue unchanged, which is likely in the short term, particularly as current vaccines against human papillomavirus cannot provide full protection against all oncogenic human papillomavirus types. If the prevalence of human papillomavirus infection is, however, significantly reduced as a result of universal vaccination, as our model predicts, then it may be possible to extend the interval between routine screens or to increase the age at which screening is first offered, as suggested in other cost effectiveness studies.^29^ ^52^ Such analyses would also need to take account of possible changes to the cervical screening programme, such as the introduction of DNA testing for human papillomavirus, as changes in the prevalence of infection after vaccination would be expected to alter the attractiveness of screening tests with improved sensitivity (which is provided by testing for human papillomavirus). It is clear that significant further work on the design of optimal screening and vaccination programmes is required, particularly if the introduction of vaccination accelerates the recent trend for the declining uptake of screening.^21^

Models are simplifications of reality and the strengths of the conclusions drawn from modelling studies depend on the reasonableness of the assumptions and parameters that make up the model. By adopting a range of assumptions and parameter estimates the approach taken here should generate more robust conclusions than can be derived from studies in which a single model structure is chosen and sensitivity analysis done on the parameter values one or two at a time. Although considerable uncertainty remains, the study also highlights the areas of research that could be taken to reduce this uncertainty. With the adoption of human papillomavirus vaccination in the UK and other countries, some of the other aspects of the clinical course of human papillomavirus should become apparent. High quality surveillance combined with mathematical models will, however, be needed to help disentangle the complex epidemiological patterns that are likely to emerge after immunisation. Refinement of these mathematical and economic models will be necessary to help optimise screening and vaccination programmes in the future.

**What is already known on this topic**

Human papillomavirus (HPV) vaccination could decrease the incidence of cervical and other cancers and anogenital warts
Existing economic analyses of HPV vaccination do not consider many of the possible sources of epidemiological and economic uncertainty or end points that vaccination may prevent

**What this study adds**

Routine vaccination of girls aged 12 combined with an initial catch-up campaign up to age 18 is likely to be cost effective in the UK
Vaccination of boys is unlikely to be cost effective
A vaccine against HPV types 16 and 18 costing £13-£21 less per dose (depending on duration of vaccine protection) would be as cost effective as a vaccine against virus types 6, 11, 16, and 18

## Notes

We thank Ruby Siddiqui for information on the literature about cost and quality of life implications of human papillomavirus associated infections, Sue Westlake and the Office for National Statistics for providing incidence data on cervical cancer, Zia Sadique for statistical advice, the panel of referees who commented on an earlier version of this work, and Kate Soldan, Nigel Gay, and Elizabeth Miller for helpful discussions.

Ethical approval: Not required.

Provenance and peer review: Not commissioned; externally peer reviewed.

## Notes

**Cite this as:** *BMJ* 2008;337:a769
